# Study of surgical indication for knee arthroplasty by cartilage analysis in three compartments using data from Osteoarthritis Initiative (OAI)

**DOI:** 10.1186/1471-2474-14-194

**Published:** 2013-06-25

**Authors:** Eiko Yamabe, Teruko Ueno, Ryo Miyagi, Atsuya Watanabe, Christine Guenzi, Hiroshi Yoshioka

**Affiliations:** 1 Department of Radiological Sciences, University of California Irvine, Irvine, CA, USA; 2 Department of Orthopaedic Surgery, School of Medicine, Keio University, Tokyo, Japan; 3 Department of Diagnostic Radiology, Cancer Institute Hospital, Tokyo, Japan; 4 Department of Orthopaedic Surgery, Teikyo University Chiba Medical Center, Chiba, Japan

**Keywords:** Magnetic resonance imaging, Knee, Arthroplasty, Cartilage, Surgical indication

## Abstract

**Background:**

Bicompartmental or unicompartmental knee arthroplasty (BKA, UKA) is currently advocated as an alternative solution to conventional total knee arthroplasty (TKA) in order to preserve bone stock and ligaments for limited osteoarthritis (OA) with intact anterior and posterior cruciate ligaments (ACL, PCL). However, the actual rate of UKA or BKA compared to TKA procedures in OA patients has not been reported. In this study, we retrospectively analyzed preoperative MRI of the knee in subjects who underwent knee arthroplasty and assessed the potential for UKA or BKA as an alternative treatment.

**Methods:**

Data were extracted from the Osteoarthritis Initiative (OAI) public use data set, which included 4,796 subjects, ages 45–79. 3.0 Tesla MRI scanners were dedicated to imaging the knees of OAI participants annually from February 2004 to March 2010. Extensive quantitative measurements of the knee MRI were performed on 87 patients who underwent knee arthroplasty during follow-up visits. We assessed the cartilage thickness and defect size in the medial femorotibial joint (FTJ), lateral FTJ, and patellofemoral joint (PFJ) as well as ligamentous injury, bone marrow edema, and subchondral cyst size from 2D coronal turbo spin echo (TSE), 2D sagittal TSE, 3D coronal T1-weighted water-excitation fast low angle shot (FLASH), and 3D sagittal water-excitation double echo steady-state (DESS) with axial and coronal reformat images.

**Results:**

Eighty-five subjects (97.7%) were subjected to TKA, while only 2 subjects (2.3%) received UKA from the OAI database. Based on the preoperative MRI findings criteria, 51 of 87 subjects (58.6%) met the indication for TKA including the 2 UKA subjects above. This rate was significantly lower (p<0.001) than the actual TKA rate received. Among 85 subjects who actually underwent TKA, 31 subjects (36.5%) and 5 subjects (5.9%) met the indication for BKA and UKA, respectively.

**Conclusions:**

Many medial or lateral compartmental OA subjects, with or without patellar compartment defects have undergone TKA. The results of this study suggest the indication for partial arthroplasty, such as UKA or BKA, may increase when cartilage in each compartment, as well as ligaments and subchondral bone status are comprehensively evaluated.

## Background

Total knee arthroplasty (TKA) replaces all three compartments of the medial and lateral femorotibial joint (FTJ) and the patellofemoral joint (PFJ), and has been considered as a conventional arthroplasty solution for osteoarthritis (OA). However, TKA may be an excessively invasive procedure for most young patients that present with isolated compartmental OA and hope to return to an active lifestyle as soon as possible. The ACL, which is a primary restraint to anterior translation of the knee, is removed during the TKA surgery. To resolve this problem, less invasive unicompartmental knee arthroplasty (UKA) with replacement of only the damaged medial or lateral compartment has recently become increasingly popular and there has been evidence that suggests that UKA, in some cases, can have benefits over TKA [1,2]. UKA enables patients to quickly return to low-impact sports with higher success rates compared to TKA [1], though it is essential to conservatively select patients who meet the criteria for UKA. UKA is indicated only for lesions that involve cartilage damage alone in a single compartment [3].

Recent cadaveric and radiographic studies of normal age-associated wear of knee cartilage demonstrate that structural changes typically progress from the medial condyle to the patellofemoral compartment [4,5]. Moreover, Rolston et al. [6] suggest that patients with the combination of medial and patellofemoral compartmental OA are more common than previously thought. With these facts taken into consideration, medial and patellofemoral bicompartmental knee arthroplasty (BKA) has also been advocated as an alternative to TKA in order to preserve bone stock for limited OA with intact anterior and posterior cruciate ligaments (ACL, PCL) and lateral compartment [6]. BKA includes the arthroplasty with a monoblock implant or the combination of UKA and patellofomoral arthroplasty (PFA).

When compared to TKA, partial knee replacement such as UKA or BKA preserves not only bone stock but also the ACL and PCL leading to nearly normal knee kinematics and higher patient satisfaction [7]. Furthermore, there have been some reports indicating UKA’s merits [1,2,8], UKA’s unchanged kinematic stability [9], BKA’s faster recoveries [7,10], and BKA’s good clinical results [6]. Improvements in patient selection criteria appear to have had the greatest impact on the recently observed favorable outcomes. However, the actual rates of UKA or BKA compared to TKA procedures in OA patients have not been reported. In the present study, we used data from the Osteoarthritis Initiative (OAI) to retrospectively analyze preoperative magnetic resonance imaging (MRI) of the knee in subjects who underwent knee arthroplasty. The purpose of this study was to examine the potential of UKA or BKA as an alternative treatment from the standpoint of preoperative MRI findings of cartilage loss, ligamentous injury, bone marrow edema, and subchondral cyst size.

## Methods

### Subjects

Data for analyses were extracted from the OAI public use data set (http://oai.epi-ucsf.org/datarelease/), where 4796 subjects (ranging in ages from 45 to 79) who had symptomatic knee osteoarthritis were included. 3.0 Tesla MRI scanners were dedicated to imaging the knees of OAI participants annually over four years of follow-up (baseline, 12-month, 24-month and 36-month) from February 2004 to March 2010. Of the 4796 subjects, there were 127 who underwent knee arthroplasty (right knee; 64, left knee; 63) during follow-up visits. We extracted and evaluated 87 subjects (right knee; 44, left knee; 43) for which both preoperative knee MRI and type of arthroplasty (TKA, BKA or UKA) received could be identified. The institutional review board at each institute participated in the OAI study approved the protocol and consent form for the OAI study. Written informed consent was obtained prior to each clinic visit. Authorization for inclusion of the participant’s study data in public release datasets was part of the consent form.

### MR sequence parameters

**Figure 1:**
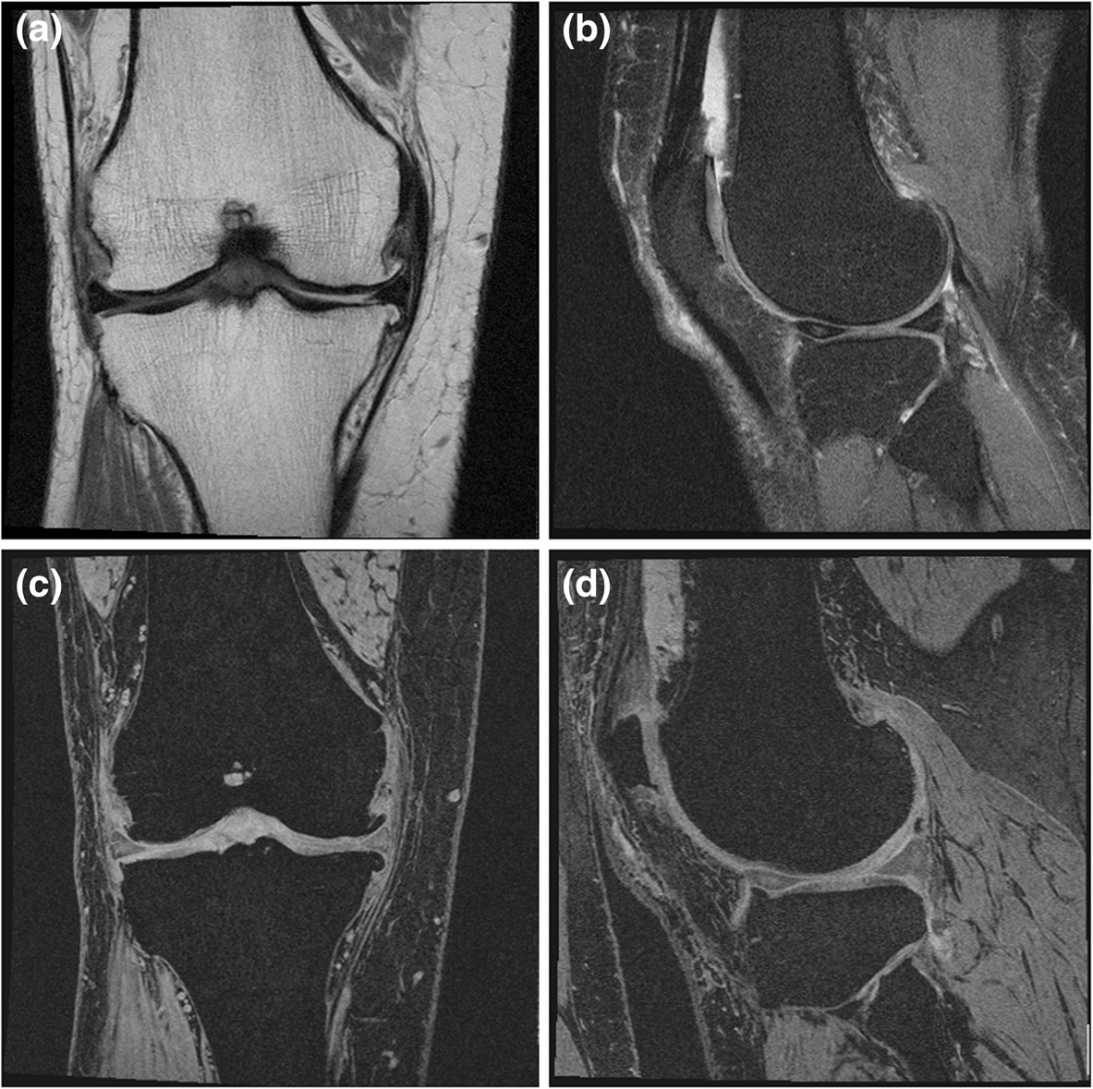
Examples of MR images; (a) 2D coronal TSE (TR/TE=3850/29 ms), (b) 2D sagittal TSE (TR/TE=3200/30 ms), (c) 3D coronal T1-weighted water-excitation FLASH (TR/TE=20/7.57 ms, flip angle=12 degrees), and (d) 3D sagittal water-excitation DESS (TR/TE=16.3/4.7 ms, flip angle =25 degrees) with coronal reformat, respectively.

Each MRI was performed according to the following sequences: 2D coronal turbo spin echo (TSE) (TR/TE=3850/29 ms, slice thickness = 3 mm, FOV= 140 mm, matrix= 307×384) (Figure 1(a)), 2D sagittal TSE (TR/TE=3200/30 ms, slice thickness = 3 mm, FOV= 160 mm, matrix= 313×448) (Figure 1(b)), 3D coronal T1-weighted water-excitation fast low angle shot (FLASH) (TR/TE=20/7.57 ms, flip angle = 12 degrees, slice thickness = 1.5 mm, FOV=160 mm, matrix=512×512) (Figure 1(c)), and 3D sagittal water-excitation double echo steady-state (DESS) with axial and coronal reformat (TR/TE=16.3/4.7 ms, flip angle = 25 degrees, slice thickness = 0.7 mm, FOV=140 mm, matrix=307×384) (Figure 1(d)).

### Assessment of MR images

**Figure 2:**
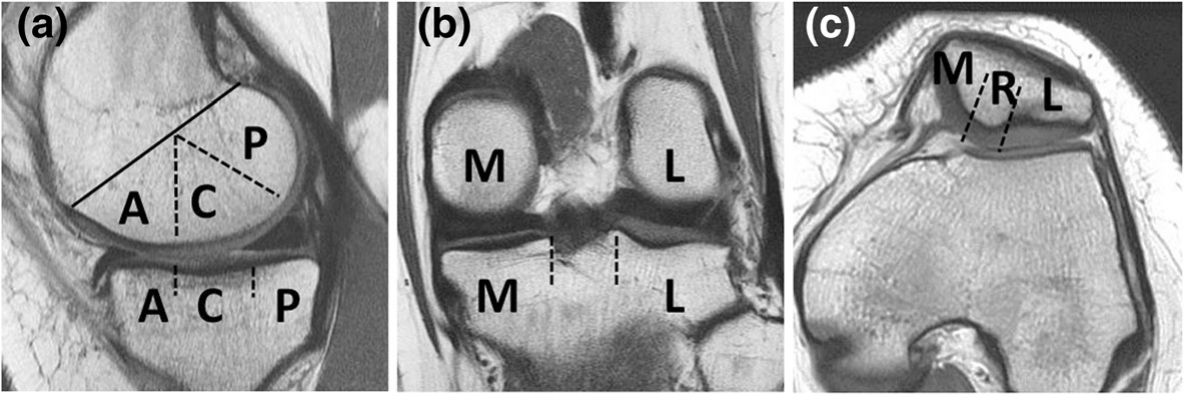
**Regional subdivision of the articular surface on (a) sagittal, (b) coronal, and (c) axial MR images.** A: anterior, C: central, P: posterior, M: medial, L: lateral, and R: ridge of patella, respectively.

We divided the three components (medial/lateral FTJ, PFJ) into three portions (Figure 2(a), (b), (c)). The medial and lateral FTJ were divided into anterior, central or posterior portions while the PFJ was divided into medial, lateral, or central portions. The cartilage thickness and defect size were assessed according to the following classifications: Grade (G) 0: normal, G1: signal heterogeneity, G2: fraying, G3: fissuring, G4: thinning<50%, G5: thinning>50%, G6: full thickness cartilage loss; and Size (S) 1: <10 mm, S2: <20 mm, S3:>20 mm [11] (Table 1). The ACL, PCL, medial collateral ligament (MCL) and lateral collateral ligament (LCL) were assessed according to the following classification: 0: normal, 1: sprain, 2: partial tear and 3: complete tear (Table 1). Bone marrow edema was classified as 0: absent, 1: mild (<1 cm in diameter), 2: moderate (1-2 cm) and 3: severe (>2 cm) (Table 1). We also classified subchondral cysts into four grades: 0: absent, 1: mild, 2: moderate and 3: severe (Table 1). Each evaluation was performed by consensus of an orthopedic surgeon and a musculoskeletal radiologist.

**Table 1:** Grading for cartilage, ligaments, bone marrow edema, and subchondral cyst

**Cartilage**	Grade 0: normal, 1: signal heterogeneity, 2: fraying, 3: fissuring, 4: thinning<50%,
	5: thinning>50%, 6: full thickness cartilage loss
	Size 1: <10 mm, 2: <20 mm, 3:>20 mm
**Ligaments**	Grade 0: normal, 1: sprain, 2: partial tear, 3: complete tear
**Bone marrow edema**	Grade 0: absent, 1: mild (<1 cm), 2: moderate (1–2 cm), 3: severe (>2 cm)
**Subchondral cyst**	1. Grade the size of each individual subchondral cyst according to the grading templates:
	Grade 1: mild (1-3 mm), 2: moderate (>3-6 mm), 3: severe (>6 mm)
	2. The sum of individual subchondral cyst grades in each compartment gives total score:
	Score 0: no subchondral cyst (0) 1: mild subchondral cysts (1–2) 2: moderate subchondral cysts (3–4) 3: severe subchondral cysts (>5)

### Assessment of optimal operative indication

We judged the optimal intervention for each subject from MR findings, such as cartilage loss, ligamentous injury, bone marrow edema or subchondral cyst size, and analyzed the difference between the actual type of arthroplasty received and the optimal arthroplasty according to our criteria. In the present study, we included greater than 50% or full thickness cartilage loss (G5+G6) in any size or at any portion of each component in the inclusion criteria for TKA. We also defined bone marrow edema and/or presence of subchondral cyst with cartilage loss less than or equal to G4 in the medial FTJ, lateral FTJ, and PFJ as medial, lateral, and PF factors, respectively. To decide the optimal arthroplasty for each subject, we used our original surgical indications (Table 2). Since severe cartilage defects in all three compartments (M+L+PF) or both medial and lateral compartments (M+L) are the best indication for TKA regardless of the presence or absence of ligamentous injuries, bone marrow edema, subchondral cyst or cartilage loss in patellofemoral (PF) compartments, we treated M+L+PF and M+L cartilage defect with G5+G6 as indications for TKA without any further investigations. Subjects with other variations of cartilage defect, including medial FTJ and PFJ (M+PF), lateral FTJ and PFJ (L+PF), only medial FTJ (M), only lateral FTJ (L), only PFJ (PF) or no cartilage defect were subsequently analyzed to decide their optimal solutions. For example, G5+G6 cartilage defects in M+PF, L+PF, M, L, or PF associated with bone marrow edema, subchondral cyst, or partial/complete ligamentous tear in the knee fall under the indication for TKA. The indication for BKA and UKA in each compartment disease is shown in Table 2.

**Table 2:** Optimal operative indications for knee arthroplasty in subjects with G5+G6 (greater than 50% + full thickness) cartilage defect

**M+L+PF**		TKA
**M+L**		TKA
**M+PF**	(+) lateral factors or ligamentous injury	TKA
	No lateral factors and no ligamentous injury	BKA
**L+PF**	(+) medial factors or ligamentous injury	TKA
	No medial factors and no ligamentous injury	BKA
**M**	(+) lateral factors or ligamentous injury	TKA
	No lateral factors and no ligamentous injury	
	(+) PF factors	BKA
	(−) PF factors	UKA
**L**	(+) medial factors or ligamentous injury	TKA
	No medial factors and no ligamentous injury	
	(+) PF factors	BKA
	(−) PF factors	UKA
**PF**	(+) medial and lateral factors	TKA
	(+) medial or lateral factors w/ ligamentous injury	TKA
	(+) medial or lateral factors w/o ligamentous injury	BKA
	No medial and lateral factors	non-operation

*TKA* total knee arthroplasty, *BKA* bicompartmental knee arthroplasty, *UKA* unicompartmental knee arthroplasty, *G* grade, *M* medial femorotibial compartment, *L* lateral femorotibial compartment, and *PF* patellofemoral compartment.

### Statistical analysis

Actual and optimal proportion of each operative procedure in these 87 subjects was statistically analyzed with Chi-square for independence test. A p-value of <0.05 was treated as statistically significant.

## Results

The type of arthroplasty received (TKA, BKA or UKA) could be identified in 87 subjects from OAI database. Of those 87 subjects, 85 (97.7%) underwent TKA and 2 (2.3%) underwent UKA. There were no patients received BKA.

### Relationship between cartilage defects and arthroplasty received

Table 3 shows the results of the relationship between knee components with G5+G6 (greater than 50% + full thickness) cartilage defect and arthroplasty. All three compartments (M+L+PF) were regarded as damaged in 33 subjects and the M+L compartments were damaged in 9 subjects. These 42 (TKA 40; UKA 2) subjects met the indication for TKA. The remaining 45 subjects (M+PF 20; L+PF 14; M 7; and L 4) who received TKA were subsequently assessed with regard to other factors such as ligamentous injuries, bone marrow edema, and subchondral cysts.

**Table 3:** Relationship between knee components with G5+G6 cartilage defect and arthroplasty received in OAI study

**Components**	**TKA**	**UKA**
**M+L+PF**	32	1
**M+L**	8	1
**M+PF**	20	0
**L+PF**	14	0
**M**	7	0
**L**	4	0
**PF**	0	0
**None**	0	0
**Total**	85	2

*TKA* total knee arthroplasty, *UKA* unicompartmental knee arthroplasty, *G* grade.

*M* medial femorotibial compartment, *L* lateral femorotibial compartment, and

*PF* patellofemoral compartment.

### Optimal arthroplasty due to the pattern of cartilage defect

**Figure 3:**
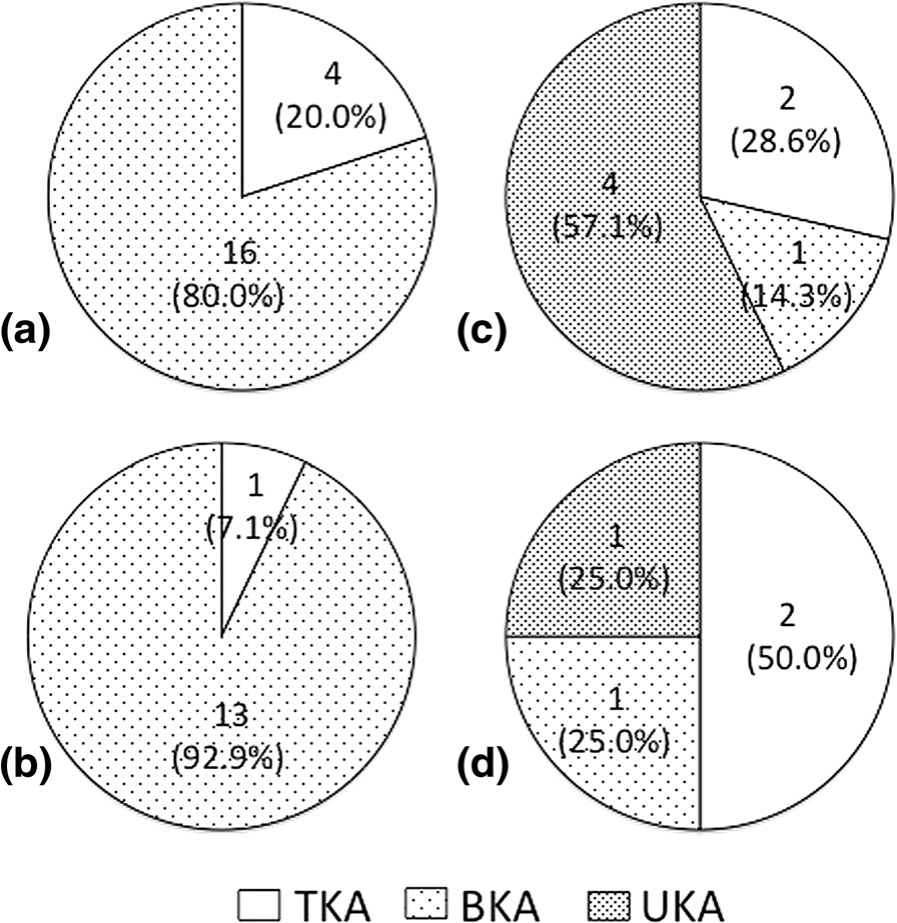
**Rate of optimal arthroplasty in the pattern of cartilage defect involvement for the subjects actually performed TKA; (a) M+PF, (b) L+PF, (c) M, and (d) L compartmental cartilage defect with G5+G6.** M: medial, L: lateral, PF: patellofemoral, TKA: total knee arthroplasty, BKA: bicompartmental knee arthroplasty, and UKA: unicompartmental knee arthroplasty.

**Figure 4:**
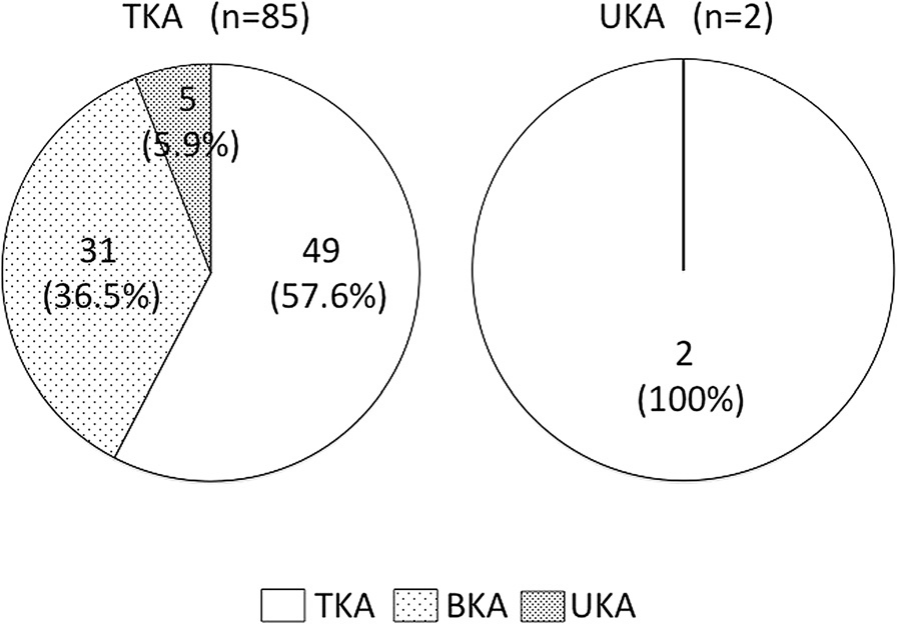
**Differences in rate of optimal arthroplasty for subjects actually performed TKA and UKA.** TKA: total knee arthroplasty, BKA: bicompartmental knee arthroplasty, and UKA: unicompartmental knee arthroplasty.

Figure 3 shows the optimal arthroplasty as determined by our operative indication in 45 subjects where TKAs were performed for M+PF, L+PF, M, or L compartmental cartilage defect. Four subjects with M+PF cartilage loss (Figure 3(a)) and one case with L+PF cartilage loss (Figure 3(b)) met the indication for TKA. The former included one subject with completely ruptured ACL and three with lateral compartmental factor, while the latter case had completely ruptured ACL and M compartmental factor. The remaining subjects with M+PF and L+PF cartilage loss met the indication for BKA. As shown in Figure 3(c, d), single compartment cartilage loss with G5+G6 demonstrated a total of 4 indications for TKA. The remaining subjects met the indication for partial arthroplasty (BKA or UKA). Two subjects for whom UKA was performed were classified as either M+L+PF or M+L compartmental cartilage loss and considered to meet the indication for TKA (Figure 4b).

### Differences in rate of operative methods between actually performed and estimated from MR images

The constellation of the above results is shown in Figure 4. Among 85 TKA subjects, TKA was indicated for 49 subjects (57.6%), BKA for 31 subjects (36.5%: 17 medial and 14 lateral), and UKA for 5 subjects (5.9%: 4 medial and 1 lateral). Among all 87 subjects, the number (or proportion) of actually performed TKA (n=85, 97.7%) was significantly higher (p<0.001) than that estimated by our surgical indications criteria (n=51, 58.6%). Though there was no subject who underwent BKA, 31 subjects (35.6%) met the indication for BKA. With respect to UKA, 5 subjects (5.7%) who underwent TKA met the indication for UKA and 2 subjects (2.3%) who underwent UKA met the indication for TKA based on MR finding criteria.

## Discussion

### Most popular surgery for knee OA: TKA

TKA is a widely used conventional solution for OA of the knee. Recently, minimally invasive surgery (MIS) has shown rapid rehabilitation and improved patient function. Indications for conventional TKA and MIS TKA are essentially the same: the presence of disabling pain associated with advanced medial and/or lateral FTJ with or without PFJ cartilage loss. However, the surgeon should have experience in conventional TKA well before undertaking MIS techniques. In addition, even with MIS, TKA is more invasive and has a longer recovery period than BKA or UKA. Removal of all three compartments of the femur, regardless of cartilage defect, may induce postoperative pain and a longer recovery period. Another serious issue with TKA is the loss of normal biomechanics. The ACL and occasionally PCL are removed during the surgery and implants need to add kinematic features that replace the function of these ligaments in order to provide original joint function. However, as a result of the restraint of implants, shearing forces will be transmitted to the bone-implant interface, which may result in subsequent loosening of the implant.

### Indication for UKA

UKA with bone-sparing and cruciate-retaining prosthesis has been developed recently as a primary intervention, especially for patients with early onset medial or lateral knee OA. UKA also has various advantages in recovery times and postoperative morbidity, and has also displayed better clinical outcomes compared with those of TKA in longitudinal studies [2,12]. Though there has been a report indicating very low influence of PF arthritis on the final outcome [13], indication for UKA must have strict criteria guidelines only allowing for single compartment cartilage damage without other factors [3]. An anteromedial wear pattern in the medial compartment is desirable because this pattern of wear correlates with a functional ACL, which is the most important factor for the success of UKA. In the present study, two subjects who underwent UKA presented with M+L+PF or M+L compartmental greater than 50% or full thickness cartilage loss and should have been considered for TKA based on the preoperative MRI findings. On the other hand, 5 subjects who met the indication for UKA based on our MR criteria actually received TKA. These cases may have other factors such as preoperative range of motion limitations and manual ligamentous laxity of the knee that could have influenced the surgeon’s choice of surgical intervention.

### Indication for BKA

BKA is also a minimally invasive arthroplasty and has been established for medial OA with patellofemoral OA without ACL and PCL damage [14,15]. Advantages of BKA in terms of recovery period, invasion and bone-sparing have also been reported. In addition, BKA has been reported to have potential as a prosthesis used in UKA revision [16]. Conversely, Morrison et al. [17] reported a higher complication rate with BKA, especially for persistent pain, and concluded that TKA was superior to BKA for medial knee OA. Unless long-term results of BKA are established, most surgeons may choose conventional TKA for the treatment of medial and patellofemoral OA. Taking these facts into consideration, however, it is unclear whether poor patient selection contributes to these complications. We think BKA should be strictly limited for medial/lateral OA with patellofemoral OA without any other factors. In the present study, a total of 31 subjects (17 for medial BKA (Figure 3(a), (c)) and 14 for lateral BKA (Figure 3(b), (d)) met the indication for BKA. Although further prospective, randomized studies on the long-term outcomes are needed in order to establish and determine the efficacy of BKA [17], there is outstanding potential for the treatment of younger or very active patients with knee OA if the selection of patients for the procedure is done properly.

### Surgical indication for knee arthroplasty based on MR finding criteria

The present study demonstrates that there are many medial or lateral compartment OA subjects with or without patellar compartment cartilage defects identified in preoperative MRIs in the OAI study. These results suggest that the indication for partial arthroplasty, such as UKA or BKA, might increase when cartilage and accompanying factors are comprehensively evaluated in each compartment preoperatively. However, 97.7% of arthroplasties performed were actually TKAs in the OAI study. This TKA rate is significantly higher than that estimated from MR findings based on cartilage loss, ligamentous injury, bone marrow edema, and subchondral cysts. Therefore, we may need to analyze and diagnose cartilage loss in each compartment more carefully and comprehensively in order to decide the most appropriate type of arthroplasty.

### Study limitations

There are several limitations in the present study. First, the sample size was small. Second, it is difficult to assess the knee joint preoperatively by MR findings alone to decide on the best surgical method as surgeons must take into account other factors such as the patient’s age, sex, weight, pain, range of motion of the knee, presence of contracture, activity, past medical history, present illness and radiological alignment. Indeed, in some cases, subjects do not complain of any pains despite terrible cartilage damage. In such a case, surgeons should not choose to do arthroplasty. Surgeons have to consider not only MRI findings but also pain localization in implant selection. Third, as this is a retrospective study using data from the OAI public use data set, we could not assess the relationship between MRI and arthroscopic findings. In addition, the evaluation and interpretation of cartilage loss depends on the surgeon’s scale, experience, and familiarity with MRI. Finally, in this study we evaluated MR findings to decide optimal arthroplasty indication by an experienced orthopedic surgeon and musculoskeletal radiologist in consensus. However, it would be more objective to have each reader evaluate MR findings independently and assess the interreader reproducibility for MR finding criteria.

## Conclusions

We retrospectively analyzed preoperative MRI of the knee using data from the OAI. Our results suggest the potential indication for UKA or BKA, which should be supported only when long-term results are expected, might increase by reviewing preoperative MRI findings including cartilage loss, ligamentous injury, bone marrow edema, and subchondral cyst.

## Abbreviations

OAI: Osteoarthritis Initiative; TKA: Total knee arthroplasty; BKA: Bicompartmental knee arthroplasty; UKA: Unicompartmental knee arthroplasty; FTJ: Femorotibial joint; PFJ: Patellofemoral joint; OA: Osteoarthritis; MRI: Magnetic resonance imaging; TSE: Turbo spin echo; FLASH: Fast low angle shot; DESS: Double echo steady-state; ACL: Anterior cruciate ligament; PCL: Posterior cruciate ligament; MCL: Medial collateral ligament; LCL: Lateral collateral ligament; M: Medial compartment; L: Lateral compartment; PF: Patellofemoral compartment.

## Competing interests

The authors declare that they have no competing interests.

## Author’s contributions

EY participated in conception and design of the study, acquisition of data, analysis and interpretation of the data, drafting the article, and critical revision of the article for important intellectual content. TU participated in acquisition of data. RM participated in acquisition of data. AW participated in analysis and interpretation of the data, and critical revision of the article for important intellectual content. CG participated in analysis and interpretation of the data, drafting the article, language edit, and critical revision of the article for important intellectual content. HY participated in conception and design of the study, acquisition of data, analysis and interpretation of the data, drafting the article, and critical revision of the article for important intellectual content. All authors read and approved the final manuscript.

## Pre-publication history

The pre-publication history for this paper can be accessed here:

http://www.biomedcentral.com/1471-2474/14/194/prepub
